# Sharing the cost? Soldier investment following colony fusion in basal termites

**DOI:** 10.1007/s00040-026-01105-w

**Published:** 2026-05-20

**Authors:** R. F. B. Padget, F. J. Thompson, R. A. Johnstone, M. A. Cant, B. Kuijper

**Affiliations:** 1 Centre for Ecology & Conservation, Faculty of Environment, Science & Economy, University of Exeter, Exeter, UK; 2 School of Biological Sciences, University of Bristol, Bristol, UK; 3 Department of Zoology, University of Cambridge, Cambridge, UK

**Keywords:** Colony fusion, Soldier production, Collective investment game, Post-fusion behaviour, Primitive termite

## Abstract

In most cooperative animal societies, interactions between groups are hostile or antagonistic. However, in a few species, groups that encounter each other can fuse together to form a new functional collective. In some ants and termites, such fusion events can be followed by changes in investment in soldiers and reproductives, for reasons that are poorly understood. Here we use a theoretical model to investigate optimal allocation to soldiers following colony fusion. Each colony can invest a proportion of its membership into the soldier caste, which provides a public benefit for the whole two-colony collective but comes at a cost to the investing colony by limiting colony growth and therefore reproduction. Our model predicts that soldier investment will typically decrease following colony fusion. However, the reported post-fusion soldier investment from empirical studies could be explained if the cost of soldier production falls following fusion. The model predicts that a colony’s reproductive output will typically decline as a result of fusion, unless the other colony makes a greater investment, or the cost of soldiers decreases sufficiently. Finally, our model predicts that smaller colonies will produce fewer soldiers than larger colonies after fusion because their lower average relatedness to the rest of the collective makes each soldier less beneficial; this means that smaller groups might, under certain conditions, gain from fusing with larger groups. Our results demonstrate two potential pathways to the evolution of group fusion in the lower termites – exploitation of additional resources or exploitation of additional soldiers (or potentially other workers). Within-group conflict, not considered in our model, may explain some discrepancies between our model and empirical reports.

## Introduction

Kinship is an important driver of cooperative behaviour in many animal societies (Hamilton 1963, 1964; Griffin and West 2003). High relatedness is particularly important in groups in which some individuals do not reproduce and instead gain indirect fitness via the reproduction of relatives. When such cooperative groups meet conspecifics from outside their group, encounters are often antagonistic, and this maintains high relatedness within groups, ensuring that unrelated groups remain distinct. This is often considered to be particularly important in eusocial societies in which most individuals never reproduce and therefore would incur an especially high cost for directing altruistic behaviour towards non-kin (Queller and Strassman 2003). However, in some ants and termites, colonies that encounter each other can behave amicably and fuse together.

In ants, some species regularly live in fused colonies, referred to as polydomous nests. Polydomy is most likely to occur between closely related colonies as a result of population viscosity, and results in several multi-queen nests sharing space and benefitting from shared resources (Robinson 2014). In some termite species, colonies also fuse, with two neighbouring colonies sharing nesting space, which often also acts as a food source (e.g. in single-piece nesting termites). Unlike polydomous ant colonies, fused termite colonies do not remain spatially distinct, and colonies that fuse are often unrelated (DeHeer and Vargo, 2008). Termite colony fusion occurs between neighbouring colonies of lower termites (those not in the Termitidae family), in which most individuals retain the ability to differentiate into reproductives. These helpers are referred to variously in the literature as ‘workers’, or more precisely as ‘pseudergates’ to differentiate them from the irreversibly sterile ‘true workers’ of higher termite species (Korb and Hartfelder 2008). Because colony fusion is more common in basal or ‘lower’ termites with totipotent pseudergates, the workers we refer to typically retain the ability to reproduce. In basal termites species, fusion between colonies is often followed by moulting of some workers into secondary reproductives, particularly if one or more primary reproductives (the king and queen) are killed as a result of intergroup conflict. Thus workers may stand to gain direct fitness benefits through inheritance of breeding status as a result of colony fusion (Johns et al. 2009).

Following fusion, workers from two colonies appear to merge into a single workforce, meaning that some colonies might benefit from the influx of workers of an incoming colony. Experiments have shown that individuals from colonies with a high proportion of workers are accepted into a new colony, while those from colonies with a high proportion of reproductives are rejected (Matsuura and Nishida 2001), suggesting that in the lower termites, some colonies (or some individuals within them) can gain fitness benefits by fusing with and exploiting the workers of an unrelated colony. However, some individuals in these fused groups of termites remain as non-reproductive workers or differentiate into soldiers, a terminal non-reproductive caste (Korb 2007; soldier reproduction only occurs in rare cases: Masuoka et al. 2021 but see Thorne et al., 2003). This presence and even proliferation of non-reproductives after groups have fused is puzzling, because average relatedness in a fused group is much lower than in an unfused colony. Additionally, it is thought that workers and soldiers provide indiscriminate benefits to all members of the group, including those that are not genetic relatives, since there is no evidence of nepotism in multi-queen groups (Atkinson et al. 2008). How such apparently altruistic behaviour is maintained in mixed-relatedness groups, in which average relatedness is considerably lower than typical social insect colonies, remains an open question but likely hinges on there being benefits to relatives in a fused group that outweigh the cost of exploitation by non-relatives.

Because inclusive fitness benefits are such an important driver of behaviour in eusocial colonies, they are often thought of as unitary organisms or superorganisms which feature adaptations at the level of the colony. At the colony level, fitness benefits are gained from producing dispersing reproductives (alates) who can go on to found new colonies. While investment in soldiers limits the maximum number of reproductives, they protect reproductives and workers or pseudergates from predation during colony growth, resulting in a greater number of successfully dispersing reproductives (Roux and Korb 2004). In this sense, the allocation by a colony of individuals to reproductive and non-reproductive castes represents an optimal life history allocation problem between investment in defence vs. reproduction (similar to that described in other eusocial species e.g. Macevicz and Oster 1976; Mitesser et al. 2007). When colonies fuse, both colonies benefit from soldiers’ defence regardless of the soldier’s original colony. However, only the colony that produced that soldier suffers the cost of the investment (fewer potential reproductives). Soldiers become a public good that both colonies can invest in (at personal cost) or benefit from. We can therefore model the investment in soldiers in a fused group as a collective investment game played by each colony in a fused group (Madgwick et al. 2018).

Here we present a two-part model of soldier investment before and after a fusion event between two lower termite colonies. First, we model soldier investment in a single, unfused colony, before using the collective investment game to model joint soldier production by colonies in a fused group. We link investment before and after fusion by modelling the initial contest between colonies to determine how initial investment in soldiers might influence the composition of the fused group.

## The model

As in some other models of intergroup conflict (e.g. Fearon 1995; Miller and Engemann 2004; reviewed in Sherratt and Mesterton-Gibbons 2013; Rusch and Gavrilets 2017), we treat the original colony as a unitary actor (we refer to the fused collective as a ‘group’ containing two colonies) and assume that there is no conflict within a colony. In modelling investment in soldiers versus reproductives, we make no assumptions about how soldiers, workers or reproductives differentiate. As such, our model is applicable to termite species in which helpers are pseudergates (typical of species that regularly fuse), or termite species in which helpers are terminally sterile. Individuals in a colony are assumed to have the same preferred outcome of an interaction, and we assume a colony-level mechanism that regulates soldier investment. Thus we assume there are no individual-level conflicts of interest over interaction outcome and soldier caste ratio. This assumption, though limiting demographic precision, gives the model flexibility to allow us to explore the effects of ecological factors on pre- and post-fusion soldier investment.

We present a two-part model, based on the collective investment game developed by Madgwick et al. (2018). Madgwick et al. (2018) derive the collective investment game to describe investment in sterile stalk cells in uni- and multi-clonal slime mould, *Dictyostelium discoideum*, aggregations. Multi-clonal *D. discoideum* aggregations and multi-family termite groups face similar investment decisions because groups contain a mixture of closely related and unrelated individuals; uni-clonal *D. discoideum* aggregations are analogous to un-fused termite colonies, living in kin-only groups, while multi-clonal *D. discoideum* aggregations are analogous to fused termite groups, living in mixed kinship groups.

**Fig. 1:**
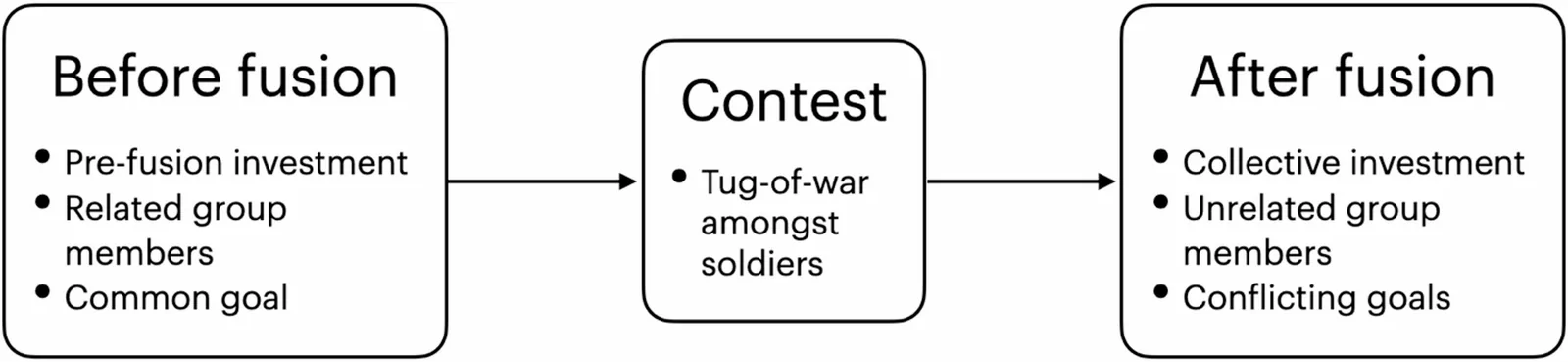
Conceptual overview of the model with characteristics of the colonies involved at each stage

Termite colony fusion occurs in stages, and the initial meeting of colonies is often characterised by intense aggression. We extend the collective investment game by modelling this initial aggression to link pre- and post-fusion soldier investment. This allows us to investigate how optimal investment might change depending on the outcome of conflict during the initial meeting, and its consequences for the composition of the fused group (Fig. 1).

### Part 1: before fusion

The first part of the model describes a single colony before it meets a second colony. We assume that any future potential for meeting another colony does not impact the investment decision of a colony at this stage because fusion is sufficiently rare that colonies do not pre-empt it. We therefore consider the cost and benefit of investing in soldiers in a colony of equally and closely related individuals. We define the cost of soldier investment as the loss in fecundity to the colony per unit investment in soldiers, and the benefit as the increased dispersal success of each reproductive (reproductive efficiency of the colony) due to the presence of a soldier. We assume a linear relationship between both cost and benefit and the number of soldiers (see Table 1 for model assumptions).

**Fig. 2:**
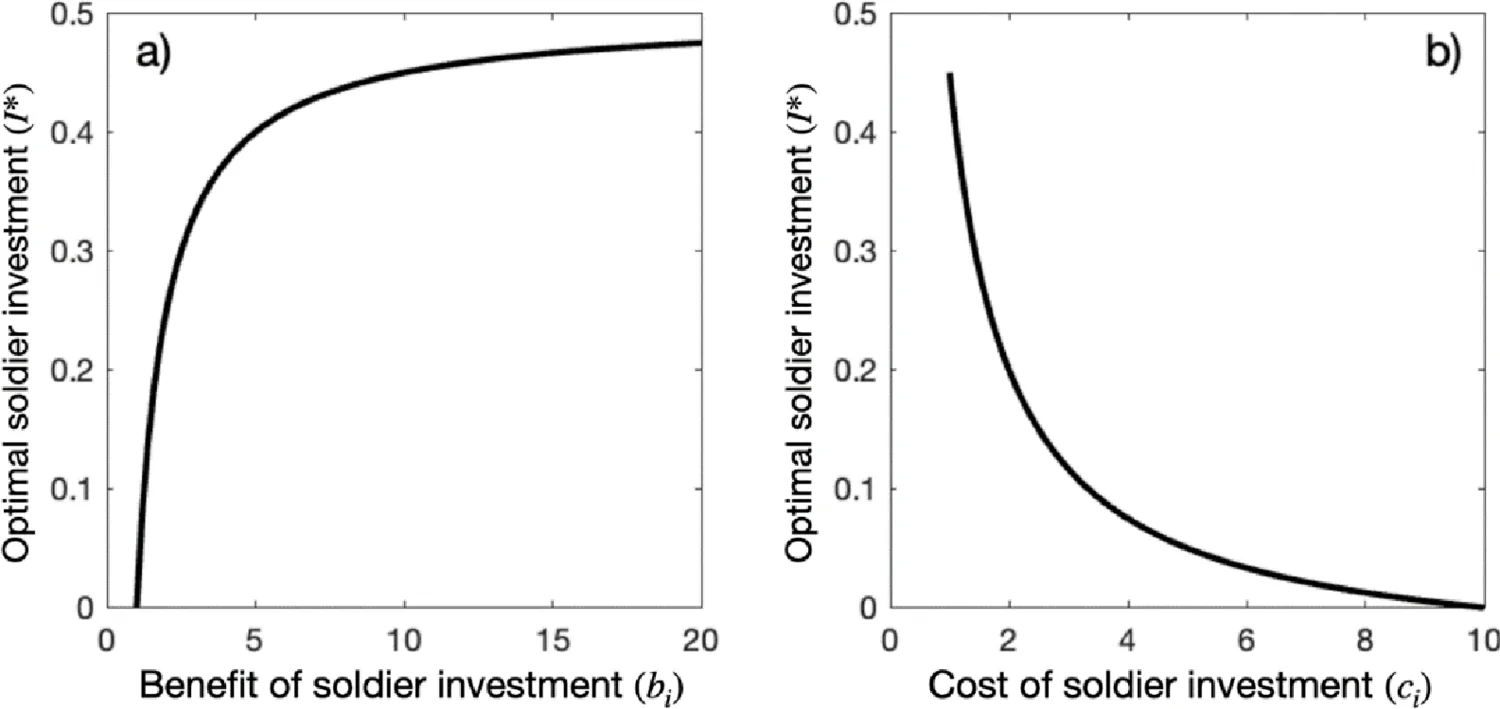
A higher benefit and a lower cost of soldiers increases the optimal level of investment in soldiers, measured as the fraction of resources invested in soldiers. Optimal investment in soldiers reaches an asymptote of (1/2)*c*_*i*_ as benefits of investment increase, so there is always lower investment in soldiers than alates. Optimal investment reaches zero when the benefit is equal to the cost. Parameters: **a** cost (*c*_*i*_) = 1; **b** benefit (*b*_*i*_) = 10

In a termite colony, fecundity is related to the number of dispersing alates as these individuals leave the nest to breed and found a new colony. For every soldier that a colony produces, there is one less potential reproductive and each soldier may also carry further costs (e.g. nutritional dependence). Fecundity (*F*) for a given colony, *i*, is therefore given by:

1 $${F_i}={\mathrm{1}} - {c_i}{I_i}$$

where *I*_*i*_ is the level of investment in soldiers as a proportion of the total possible investment and *c*_*i*_ represents the cost per unit investment in soldiers. The subscript *i* refers to the *i*th colony; there is only one colony in this part of the model, but we include *i* here for consistency with later parts of the model. Fecundity is bounded at 0 and 1 (inclusive), such that the maximum fecundity, 1, occurs when there is no investment in soldiers. Soldiers benefit the colony by providing defence, which allows the alates to develop and disperse (Roux and Korb 2004). The success of alates for colony *i* (*S*_*i*_) is given by:

2 $${S_i}={\mathrm{1}}+{b_i}{I_i}$$

where *b*_*i*_ represents the increase in alate survival conferred per unit investment in soldiers. Following from optimal brood size models (Smith and Fretwell 1974), we can multiply fecundity and survival to give colony-level reproductive output, which we can interpret as a measure of fitness (*W*_*i*_), given by:

3 $${W_i}=({\mathrm{1}} - {c_i}{I_i})({\mathrm{1}}+{b_i}{I_i}),$$

such that the minimum ‘fitness’, 0, occurs when *I*_*i*_ is 1 (i.e., there is total investment in soldiers). The proportion of resources allocated towards soldiers that maximises the fitness function for a given cost and benefit we term optimal soldier investment. Optimal soldier investment for colony *i* ($I_{i}^{{\mathrm{*}}}$) can be found by differentiating the fitness function and finding the investment when d*W*_*i*_/d*I*_*i*_ = 0. This gives:

4 $$I_{i}^{{\mathrm{*}}}=\frac{1}{2}\left( {\frac{1}{{{c_i}}} - \frac{1}{{{b_i}}}} \right)$$

(Madgwick et al. 2018), where $\:{I}_{i}^{*}$= max(*x*, 0), because soldier investment $\:{I}_{i}^{*}$ cannot be negative. As the benefit of investment in soldiers (*b*_*i*_) increases, optimal investment increases up to an asymptote of $\:\frac{1}{2}{c}_{i}$ (Fig. 2a). By contrast, as the cost of soldier production increases, optimal investment decreases to zero (Fig. 2b).

### Conflict

Part 1 of the model presented here describes a single colony’s investment in soldiers, which is a costly investment that ultimately aids success of dispersing reproductives. We now move on to model an encounter between two colonies, which is typically initially aggressive. The outcome of this aggression determines the composition of the subsequent fused group, which is important because this affects the average relatedness of a fused group (Johns et al. 2009). Since termite soldiers are the primary aggressors in contests, investment in soldiers is likely the main predictor of the outcome of this initial meeting. However, other workers might also participate in aggressive encounters (Thorne 1982), so we also include the relative size of a colony to model the role of non-soldier workers. The size term (*s*) therefore represents the relative number of workers; our model does not make the assumption that termites can identify relative colony size, though there is some evidence of this (e.g., Evans et al. 2005; Evans et al. 2009). We implement size and soldier number as independent parameters, though in nature, the number of soldiers and workers is likely to be correlated (i.e., a larger colony is likely to also have a greater number of soldiers) depending, for example on the resources available to a colony. We model worker and soldier production differently because the castes are dissimilar in a number of important ways. For example, workers provide food for both themselves and others in the colony, meaning that they do not incur the same energetic costs as soldiers. Particularly relevant for this model is that workers are not terminally non-reproductive, therefore the production of workers does not reduce the potential production of reproductives (in fact reproductives emerge via the pseudergate worker stage).

We use a tug-of-war contest success function (Hirshleifer 1989; Reeve et al. 1998; Taborsky et al. 2021) to determine how differential investment in soldiers might affect the relative proportion of each initial colony in a fused group. We can say that the representation of colony *i* (*R*_*i*_) in the fused group is given by its soldier investment relative to that of colony *j*, scaled by the difference in size (*s*) between the two groups.

**Fig. 3:**
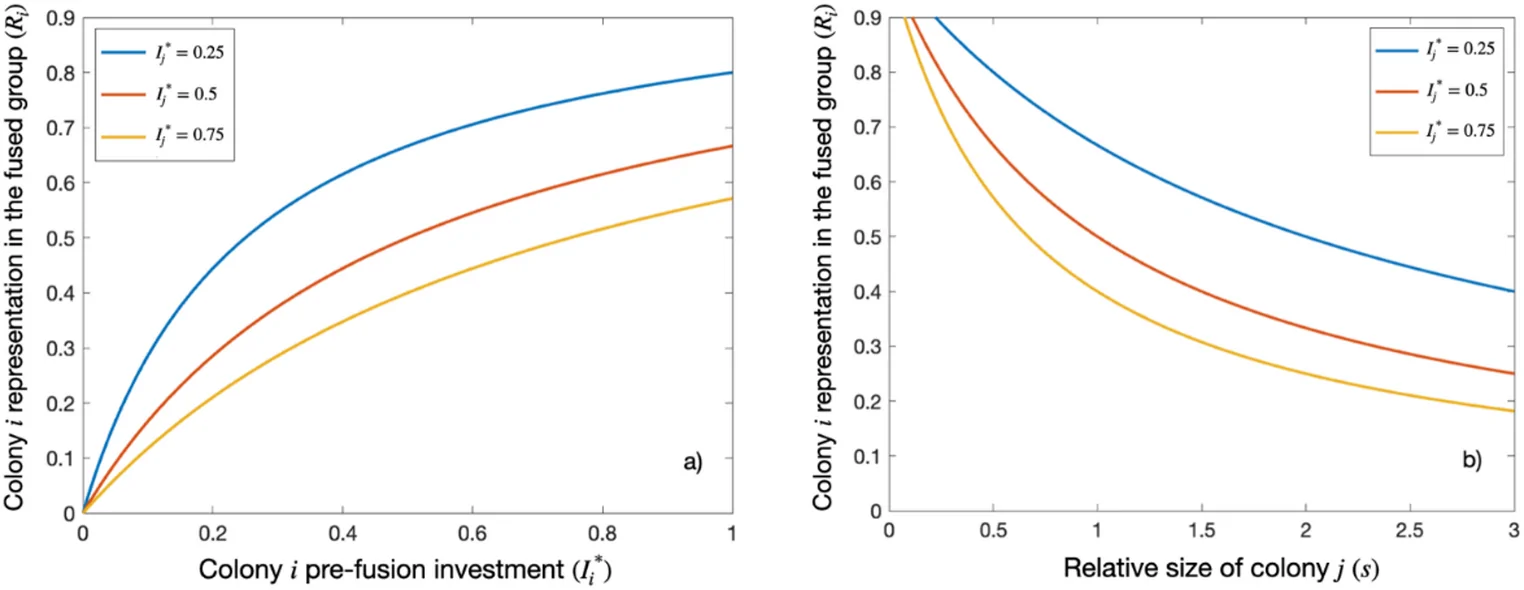
Greater investment in soldiers before fusion and smaller opponent size result in higher representation in the fused group. **a** Colonies that invest more in soldiers before fusion are better represented in the fused group, but higher pre-fusion investment by the other colony decreases the rate at which investment ‘pays off’ and results in more equal overall representation; each line represents a different level of investment by the other colony: yellow shows high investment by the other colony; red, an intermediate investment; and blue a low investment by the other colony. Other parameters: *s* = 1. **b** The larger colony *j* relative to colony *i* (*s*), the lower the representation of the colony *i* in the fused group. When colony *j* makes a lower investment in soldiers before fusion (blue line), this means that colony *i*’s representation is higher in the fused group than when colony *j* makes an equal (red) or higher (yellow) investment. Other parameters: *I*_i_*=0.5 (Color figure online)

Assuming that both colonies invest optimally given the environmental conditions that each experiences before they meet, the proportion of the fused group that colony *i* makes up is given by:

5 $${R_i}=\frac{{I_{i}^{*}}}{{I_{i}^{*}+s~I_{j}^{*}}}$$

where $\:{I}_{i}^{*}$and $\:{I}_{j}^{*}$ are the (pre-encounter) optimal investment levels of two colonies *i* and *j*, respectively (Eq. (4)); *s* is the relative size of colony *j*, where a value of 1 would mean that groups are equally sized (*s <* 1 denotes that colony *j* is smaller than colony *i* and *s >* 1 denotes that colony *j* is larger than colony *i*). Because we assume that there are exactly two interacting groups, the representation of the second colony, *j*, in the fused group is $\:{R}_{j}=1-{R}_{i}$. Representation in the fused group affects average relatedness in the fused group: assuming that pair-wise relatedness is equal within a group, but zero (or at least equally low) between individuals of different groups, representation of colony *i* in the fused group can be used as a proxy for the average relatedness of an individual from colony *i* to the whole two-colony group. We assume that the benefits soldiers provide are available indiscriminately to its original colony and the other colony in the group (Table 1), thus the average relatedness is equivalent to the probability that a soldier benefits its own kin. Figure 3a and b illustrate the effects of relative investment and size on representation in the final group for some example parameters.

### Part 2: after fusion

Following the initial aggression, colonies may fuse. All soldiers now benefit both colonies, regardless of which colony they originated from (i.e. they become a public good). This assumption is broadly appropriate because soldiers promote a general reduction in predator presence (Traniello 1981) and improve foraging efficiency in foraging species (do Sacramento et al. 2020), benefitting both colonies (Table 1). We assume that each original colony retains control over its soldier investment even after fusion, and thus can optimise its own soldier investment in the context of the collective. Each colony faces a dilemma about how much to invest given that some of this investment could be ‘wasted’ if it benefits unrelated individuals from the other original colony. Additionally, if both colonies over-invest in soldiers, the group might have redundant soldiers that do not provide sufficient additional benefit to outweigh the cost in lost fecundity. However, if colony *i* does not invest in soldiers, it risks leaving alates undefended if the other colony behaves similarly. Each colony therefore faces a dilemma centred on minimising lost reproductive opportunity while avoiding failure to invest sufficiently in soldiers (this is similar in structure to biparental care models e.g., Houston and Davies 1985). For simplicity, we ignore the presence of pre-fusion soldiers, but their effect indirectly carries over through a colony’s representation in the fused group.

We assume that colony-mate (equivalent to kin) discrimination in the benefits that soldiers provide is absent after fusion has occurred, so the probability that a soldier produced by one colony will help an alate from the same colony (i.e. a genetically related alate) is proportional to the representation of that colony in the group. In case $\:{I}_{i}^{{\prime\:}}$ and $\:{I}_{j}^{{\prime\:}}$ reflect the post-fusion investment in soldiers (distinct from pre-fusion investment *I*_*i*_ and *I*_*j*_) by colony *i* and *j* respectively, we can extend the previous, single-colony fitness function in Eq. (3) to include the *i*th colony’s representation in the fused group:

$$W_i = (1-c_i'I_i')\left(1 + b_i' \:{\sum\:}_{k=1}^2R_k(I_1, I_2)I'_k\right)$$

where the first term reflects how the investment in soldiers by the *i*th colony comes at the expense of its production of alates, which is identical to Eq. (1). The second term reflects how the development and dispersal of these alates is positively affected by soldiers defending the nest, which is now a function of total investment in soldiers $\:{\sum\:}_{k=1}^{2}{R}_{k}{I}'_{k}$ by both fused colonies (cf. Equation (2)). We can then derive optimal investment in soldiers of both colonies by solving $\:\mathrm{d}{W}_{i}/\mathrm{d}{I}_{i}^{{\prime\:}}=0$ and $\:\mathrm{d}{W}_{j}/\mathrm{d}{I}_{j}^{{\prime\:}}=0$. The non-closed-form solutions of post-fusion soldier investment (*I*_*i*_^’^, *I*_*j*_^’^ ) of both colonies are then given by:

7 $$~I_{i}^{\prime }=\frac{1}{2}\left( {\frac{1}{{c^{\prime}}} - \frac{1}{{b^{\prime}{R_i}}} - \frac{{I_{j}^{\prime }\left( {1 - {R_i}} \right)}}{{{R_i}}}} \right)$$

8 $$I_{j}^{\prime }=\frac{1}{2}\left( {\frac{1}{{c^{\prime}}} - \frac{1}{{b^{\prime}\left( {1 - {R_i}} \right)}} - \frac{{I_{i}^{\prime }{R_i}}}{{(1 - {R_i})}}} \right),$$

where $c^{\prime}$ is the post-fusion cost and $b^{\prime}$ is the post-fusion benefit of soldiers. The first term, $\frac{1}{{c^{\prime } }}$, represents the cost of soldiers in lost reproductive opportunity; this term is the same for both colonies, because they now share resources and so the cost of soldiers is assumed to be identical. The second term represents the benefit per unit of soldier investment, multiplied by the probability that the benefit will go towards the colony that produced that soldier. The final term represents the benefit to colony *i* of soldier investment by the other colony. Equations (7,8) can then be solved to yield the optimal solutions of post-fusion soldier investment ($\:{I}_{i}^{*{\prime\:}},{I}_{j}^{*{\prime\:}})$.

In a fused group, the success of alates from each colony depends on the combined investment of both colonies in the group. Because we assume each colony invests proportionally to their representation in the group, the joint investment of the group (*I*_*G*_) is defined as the weighted average level of investment by each colony (*I*_*G*_ = 𝚺*I*_*i*_^’^*R*_*i*_). This means that post-fusion fitness of the colonies (*W*_*i*_^’^, *W*_*j*_^’^) depends on the group investment and the colony’s own investment:

9 $$W_{i}^{\prime }=\left( {1 - c^{\prime}I_{i}^{\prime }} \right)\left( {1+b^{\prime}{I_G}} \right).$$

### Model interpretation

With this model, we investigate the investment in soldiers made by colonies of termites in a fused group. We choose parameter values in figures to exemplify their effects, but these results hold qualitatively across a range of values. Firstly, we explore how optimal investment in soldiers changes following fusion. We then investigate how post-fusion optimal investment is influenced by representation in the group and the other colony’s investment, and how this is constrained by the cost and benefit of soldier production in a fused group. Using the measure of fitness defined above (Eq. (9)), we then examine how different outcomes of fusion, in terms of group composition, can affect colony-level fitness.

**Table 1:** Assumptions made in the model and their justification

Assumption	Justification
Linear relationship between cost and benefit of soldiers and their production	We assume that colonies are not under nutritional constraint such that the cost of additional soldiers does not accelerate. The per capita benefit from soldiers could be constant in scenarios in which there are high numbers of predators in an area
Individuals in a colony share a preferred outcome of an encounter	Individuals in a colony are closely related and their fitness outcomes are therefore linked
There is a mechanism by which individuals in a colony collectively make a decision on behaviour	Caste differentiation appears to be dependent on caste ratio in the colony (Noirot 1991). Muller and Korb 2008 show that sex ratio allocation of soldiers is consistent with colony-level control
Only two groups interact	Fused groups comprising more than two groups appear to be rare (Howard et al. 2013)
Soldiers do not favour kin with the benefits that they provide	Soldiers likely result in a general reduction in predator presence, which benefits all individuals in the area (e.g., *Nasutitermes* soldiers deter ants on foraging trips; Traniello 1981); soldiers improve foraging efficiency (do Sacramento et al. 2020) and appear to provide social buffering against stress induced by competitors (Tian et al. 2017)
The costs and benefits are equal for each colony in a fused group	Colonies now share space and resources
Colony size (*s*) is independent of investment in soldiers	Applies when allocation to soldiers and alates does not limit helper production (Roux and Korb 2004)
Colonies maintain their own agency after fusion	High genetic relatedness within colonies means that fitness interests of individuals in a colony within a fused group are generally more closely aligned to those of their colony-mates than their non-colony-mates in the wider fused group, meaning that it is likely to be beneficial for colonies to maintain agency in a fused group. Mechanistically, top-down regulation of caste differentiation is driven by hormones secreted by reproductives. For this to impact that reproductive’s colony alone, there would need to be kin discrimination or spatial clustering of colonies

## Results

### Optimal investment in soldiers before and after fusion

**Fig. 4:**
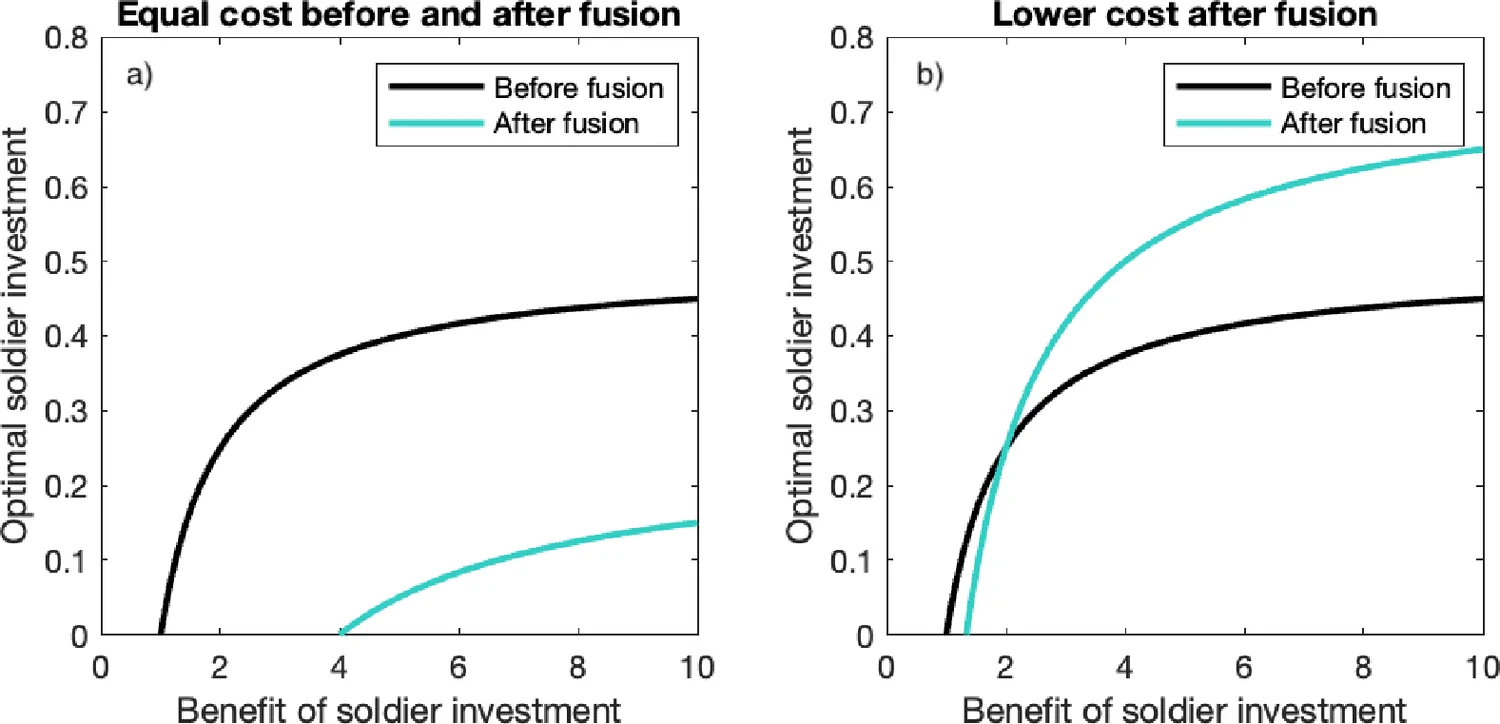
Optimal soldier investment is usually lower but can be higher after fusion, provided the cost is sufficiently reduced. Both lines show how optimal soldier investment changes with the current benefit of soldiers; the black line shows the pre-fusion optimal investment, the blue line shows post-fusion optimal investment. When all other parameters are equal, soldier investment is lower after fusion (**a**). However, when the cost of soldiers (in terms of lost reproductives) is sufficiently reduced, post-fusion soldier investment can be higher than pre-fusion soldier investment (**b**). Parameters shown: before fusion: *c = 1*; after fusion: *c’ = 0.5*,* R*_*i*_ *= 0.5*,* I*_*j*_*’ = 0.5*)

The relationship between the cost and benefit of soldier investment and the optimal soldier investment remains similar before and after fusion (optimal investment increases with benefit and falls with cost). However, optimal investment is typically lower following fusion than before because investment depends on representation in the group, which necessarily falls after fusion (Fig. 4a). However, if the relative cost of soldier production greatly falls after fusion, then it is possible that soldier production can be higher after fusion than before (Fig. 4b).

**Fig. 5:**
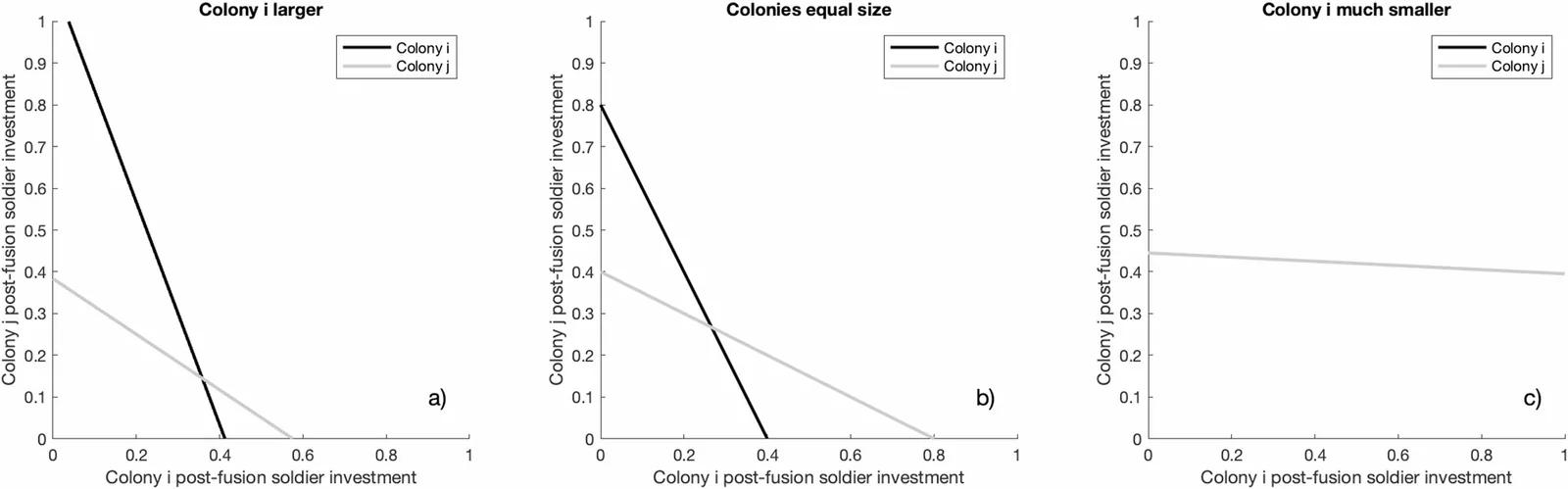
How post-fusion optimal soldier investment of both colonies changes with post-fusion soldier investment of the other colony, depending on the relative size of the colonies. The lines show the best response of one colony to the other’s soldier investment; the lines cross at the Nash equilibrium where both colonies are investing optimally in soldiers given the investment of the other colony; each panel shows a different relative size of original colony (*s*), thus relative representation in the fused group (pre-fusion optimal investment is assumed to be equal). When colony *i* is larger than colony *j* (e.g., *s = 0.75*; panel **a**) colony *i* should invest more than colony *j*. When colonies are equal sizes and so equally represented in the fused group, they should invest equally in soldiers (*s = 1*; panel **b**). When colony *i* is much smaller than *j* (e.g., *s = 10*; panel **c**), it can be optimal for it to invest nothing after fusion, while colony j continues to invest. The pre- and post-fusion costs and benefits of soldier investment are equal for both colonies. Parameters shown: *b*_*i*_ *= b*_*j*_ *= 10*; *c*_*i*_ *= c*_*j*_ *= 1*; *Ii = Ij = 0.45*

Following fusion, a colony’s optimal investment depends on the investment made by the other colony: when colony *j* makes a greater investment, colony *i* should make a lower investment, and vice versa (Fig. 5). A colony’s optimal soldier investment following fusion also depends on its representation in the fused group, determined by the relative size of each colony before fusion (*s*), with larger – thus better represented – colonies predicted to invest more than smaller colonies (Fig. 5a, c).

### Fitness outcomes of colony fusion

**Fig. 6:**
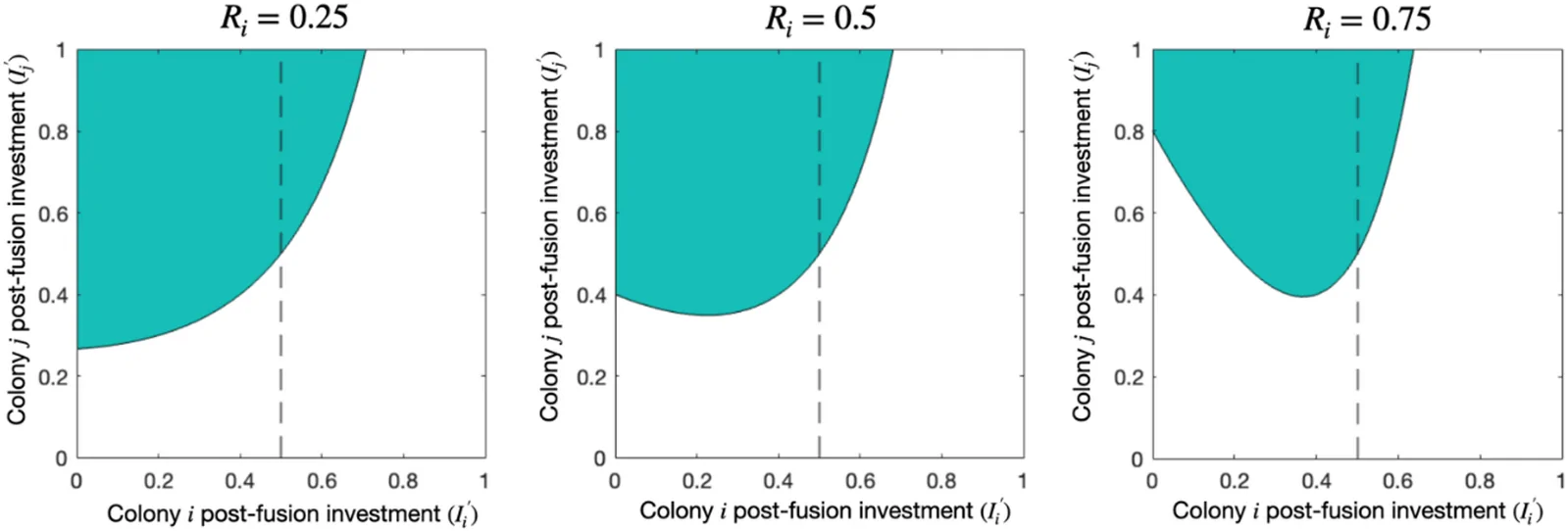
The effect of fusion on fitness for different levels of post-fusion investment by colony *i* (x axis) and colony *j*(y axis). The green region shows where the fitness of colony i is higher after fusing with colony j than before; the dashed line shows colony i’s pre-fusion investment; each panel shows a different level of representation of colony i in the fused group. If colony i is the minority colony (left-most panel) in the group (i.e., because it is the smaller original colony), it can gain fitness after fusion for a greater region of the parameter space than if the colonies are equally represented (middle panel) or if colony i is the majority colony (right-most panel). While minority colonies can gain after fusion by greatly reducing their investment in soldiers, an equally sized or majority colony can become worse off after fusion if it reduces its investment too much without sufficient compensation by the other colony. A colony can gain from fusion by reducing its investment, but even if a colony invests more in soldiers after fusion than before (green areas to the right of the dashed line), provided that the other colony also invests sufficiently, it can still be beneficial to fuse (Color figure online)

Whether or not colonies become fully integrated, there remains a genetic distinction between the two colonies, which means that it is useful to characterise the post-fusion fitness of each colony separately. The fitness impact of colony fusion depends on the relative soldier investment made by each colony, its pre-fusion investment and its representation in the fused group (Eq. 9). If a colony reduces its investment after fusion, it can gain from fusion, even if the other colony’s investment in soldiers is lower than its own (Fig. 6). If colony *i* invests the same before and after fusion, then colony *j* must invest more in order for colony *i* to make fitness gains from fusion, and if colony *i* invests more in soldiers following fusion then then colony *j* must make a much higher investment in order for colony *i* to benefit from fusion. The impact of the other colony’s investment is greater for colonies that are less well-represented in the fused group; this means that a smaller colony could lose out more than a larger one if the larger colony makes little investment in soldiers. However, it also means that smaller colonies have more to gain from fusion, and are more likely to benefit from fusion even if they invest more after fusion than before (Fig. 6).

## Discussion

Here we developed a game theoretic model of collective investment in soldiers by a colony of termites following fusion with another colony, after which the benefits, but not costs, of investment are shared by the two colonies. This model predicts that optimal investment in soldiers typically decreases following fusion, but that if the costs of soldiers are sufficiently lower in the fused colony (or benefits are higher), then optimal soldier investment can increase following fusion. The fitness of a colony can either increase or decrease following fusion depending on the relative soldier investment made by each colony and a colony’s representation in the fused group. If one colony makes a much lower investment than the other, then its fitness can increase after fusion. However, if a colony makes a much higher investment than the other, then its fitness can decrease. Additionally, smaller colonies, which make up a minority proportion of a fused group are predicted to increase their fitness to a greater degree and for a greater range of the parameter space than larger (majority) colonies are. Smaller minority colonies are also predicted to invest less, suggesting that smaller colonies might be able to take advantage of larger colonies and their soldiers after fusion.

### Optimal investment in soldiers before and after fusion

Some empirical evidence suggests that colonies in fused groups continue to produce soldiers (Howard et al. 2013). Our model predicts that a colony’s optimal investment can be equal or higher following fusion under two scenarios: (1) when the other colony makes little or no investment (e.g., Fig. 5), and (2) when the cost of soldier production sufficiently decreases after fusion (e.g., Fig. 4). A minority colony is predicted to invest very little, so a colony that finds itself in the majority in a fused group might be predicted to optimally invest more in soldiers than it did before fusion to compensate for this. This is tentatively consistent with empirical observations by Johns et al. (2009) who find that most of the soldiers in a fused group originate from the majority colony. However, the skew in soldier origins is slight in this study, and there is only limited evidence that these soldiers differentiated after the fusion event (therefore, the result may simply be a numerical outcome of different-sized colonies). Further empirical work that can identify the origin colony of soldiers that differentiate after fusion would help to test the prediction that smaller colonies have more to gain from fusion. Similarly, while there is some evidence of kin and nestmate recognition in termites (Kaib et al. 1996; recently reviewed in Korb 2025), more experimental tests of the extent of this recognition would help to determine how widespread mechanisms are that allow termites to respond to relatedness heterogeneity in such a way that would allow colony-level control of soldier differentiation.

Following fusion, the model predicts that a colony should invest more than it did before fusion if the cost of soldiers decreases or the benefit increases from a low baseline. The benefit that soldiers confer could increase following fusion if larger groups are more vulnerable to predation, for example. Alternatively, if the benefit from soldiers accelerates with the number of soldiers then any additional soldier investment could have a disproportionate positive effect on colony fitness. However, the benefit of soldiers could also decrease because colony fusion removes a potential competitor from the environment, making another violent encounter less likely; the larger size of the fused group also means that future conflict will likely be less costly. Relative costs of soldier production could fall after fusion if there are more resources available per capita than before fusion, as resource availability can limit soldier number (Song et al. 2006). Although an increase in resource availability does not change the amount of resource required to produce a soldier, it means that there are more resources available to potential reproductives, thus there is less pressure on the trade-off in resource allocation to soldiers vs. reproductives, which is the ultimate ‘cost’ of a soldier. Resource availability might be relevant for colonies of single-piece nesting termites, which could gain access to important, patchily distributed resources by fusing (Shellman-Reeve 1994). Single-piece nesting colonies spend their entire colony lifespan inside a single piece of dead wood; if a colony were to fuse with another in the same piece of wood, they would gain access to the wood resource that the other colony was previously occupying, providing the benefit of additional resource (i.e., where the colony originally could use only a share of its log, the fused colony can now use the whole log). Because there is no other way to gain additional resources (e.g., moving to another log is not an option), this resource acquisition via fusion could determine the relative cost of soldiers in these species. However, a positive relationship between colony fusion and access to resources is less likely to occur in nomadic and foraging groups because these groups are not as spatially constrained in their resource use. Our model would therefore predict that single-piece nesting termites are more likely to increase soldier production following fusion than nomadic or foraging termites.

### Fitness outcomes of colony fusion

In most post-fusion scenarios, a colony’s best outcome in terms of producing successful alates (i.e. fitness) is when it avoids investing in soldiers but can exploit the investment of the other colony. This prediction is consistent with evidence that *Reticulitermes speratus* colonies are more likely to fuse with another colony that has a high proportion of workers (compared to nymphs, a pre-reproductive caste), and are aggressive towards colonies with a lower proportion of workers (Matsuura and Nishida 2001). This could indicate that colonies can indeed gain from the additional workers produced by the other colony to provision their own nymphs (Matsuura 2001) and are unwilling to absorb nymphs, whom they must provision at a cost. This could also extend to exploiting other colonies’ soldiers, but as yet, there is no empirical evidence of such exploitation.

Alternatively, a colony could gain fitness benefits from fusion if the costs of soldier production decrease or the benefits increase as a result of colony fusion. Lower costs enable higher investment in soldiers without loss of reproductive investment, providing greater benefit to the colony. Alternatively, a higher benefit from soldiers means that less investment is needed to gain the maximum benefit, allowing greater resource allocation towards reproductives. The cost and benefit of soldier production is likely to change for a colony following fusion, because colonies have access to greater resources, however these resources must be shared amongst a larger group of individuals. More empirical investigation into resource efficiency in termite colonies before and after fusion, and its potential impact on caste differentiation is warranted.

The model predicts that there are two ways in which group fusion could be beneficial to a colony: (1) if the relative cost of soldiers decreases following fusion, meaning that soldier investment is more efficient (i.e., the same number of soldiers can be produced for lower investment); or (2) if the other colony’s investment in soldiers can be exploited. These two options could be relevant to termite species of different nesting types. Fused colonies of single-piece nesting termites might be more likely to gain access to patchily distributed resources, which would allow them to invest more efficiently in defensive soldiers following fusion (Shellman-Reeve 1994). Conversely, foraging termites are less likely to benefit from gaining resources following fusion, and are thus predicted to be less likely to invest in soldiers following fusion (although foraging termites could benefit from another colony’s worker caste; Matsuura and Nishida 2001). Comparative investigation of post-fusion investment in soldiers across termites of different ecologies would help to test this prediction.

Our model also shows that representation in the fused group can be an important factor in determining whether a colony makes fitness gains or losses from fusion. The fitness of a colony with lower representation in the fused group is affected to a greater extent by the soldier investment of the other colony than the fitness of a colony with high representation. While this means that a minority colony in a group can suffer greater fitness losses if the other colony does not invest sufficiently, it also means that a minority colony can make greater fitness gains from the investment by the other colony. Representation in a fused group is likely to be primarily determined by the relative sizes of the colonies before fusion, meaning that a smaller colony might have greater opportunity to benefit from fusion with a large colony than a large colony has to gain from fusing with a small colony. The outcome of different-sized colonies meeting is therefore likely to depend on the information that each colony has about its own size relative to that of an interacting colony, and each colony’s ability to respond to this information. Information about relative colony size could be manipulated empirically by staging interactions in different settings with different colony sizes, for example petri dishes in which individuals are able to interact more freely could give individuals more information about the other colony compared to narrow tubing in which meetings between individuals are sequential.

In this model, we considered the original termite colony of related individuals to be a unitary actor both before and following fusion, however there are likely to be differences in potential fitness outcomes of fusion for different castes. For example, soldiers have no future direct fitness opportunities, but in lower termites, pseudergates can and do gain direct fitness benefits from colony fusion (Johns et al. 2009; Korb and Roux 2012). This could result in within-colony conflict after fusion (Korb 2025). For example, there is evidence that the presence of hormones from reproductives can trigger soldier differentiation (Luscher 1960; Maekawa et al. 2012). When pseudergates moult into reproductives following fusion, this could increase the concentration of the hormones involved in reproductive inhibition and soldier differentiation, resulting in additional soldiers developing. If these soldiers do then indiscriminately benefit the whole fused group, this represents a potential mechanism by which within-colony conflict could result in between-colony cooperation.

Our model assumes that both colonies have perfect information about their own and the other colony’s post-fusion costs and benefits; modelling frameworks that incorporate information and uncertainty (e.g., Bayesian or sequential games) could allow for analysis of negotiation of soldier investment over a finer temporal scale than we modelled here. Modelling individual-level fitness decisions (e.g., the volunteer’s dilemma), in which individuals (rather than colonies) choose whether or not to invest in a public good could also offer insight into how the phenotypic heterogeneity typical of termite (and other social insect) colonies might play a role in the differentiation of a non-reproductive caste in groups of mixed relatedness (Diekmann 1985; Hauert et al. 2002; Archetti 2009). Experimental manipulation of hormones that regulate caste differentiation during fusion could also help to reveal the proximate mechanisms that underpin caste differentiation during colony fusion and thus facilitate this example of intergroup cooperation.

## Conclusions

Our model shows that fusion between colonies, which is common in basal termites, can have profound impacts on the reproductive division of labour within colonies. The dilution of relatedness following fusions favours reduced investment in soldiers. By contrast, increased colony size can favour improved resource acquisition and greater production of soldiers after fusion. The potential fitness benefits of fusion are likely to differ between termites of different ecologies, therefore comparative analysis of fusion events in termites will likely help to explain this counter-intuitive phenomenon.
